# *Staphylococcus sciuri* causes disease and pathological changes in hybrid sturgeon *acipenser baerii* × *acipenser schrencki*

**DOI:** 10.3389/fcimb.2022.1029692

**Published:** 2022-10-06

**Authors:** Mengwei Zhang, Mingyang Xue, Zidong Xiao, Wei Liu, Nan Jiang, Yan Meng, Yuding Fan, Xiaoling Liu, Yong Zhou

**Affiliations:** ^1^ Department of Aquatic Animal Medicine, College of Fisheries, Huazhong Agricultural University, Wuhan, China; ^2^ Yangtze River Fisheries Research Institute, Chinese Academy of Fishery Sciences, Wuhan, China

**Keywords:** hybrid sturgeon, *staphylococcus sciur*, intestinal microorganisms, hematological parameters, histopatholog

## Abstract

Hybrid sturgeon is the main species of sturgeon cultured in China, with the advantages of a fast growth rate, early sexual maturity, fertile offspring, and more stable genetic traits. In May 2021, a large number of deaths characterized by superficial hemorrhage and liver damage occurred in a sturgeon farm in Yichang, Hubei Province, which posed a significant risk to hybrid sturgeon captive breeding. We isolated a pathogenic bacterium named D-59 from the diseased sturgeon with apparent symptoms. The pathogen was identified as *Staphylococcus sciuri* using 16S rRNA gene phylogenetic analysis combined with biochemical identification. Regression experiments showed that D-59 exhibited clinical signs similar to those of diseased sturgeon in the farm after intraperitoneal injection into hybrid sturgeon. High-throughput sequencing of gut microbes in D-59-infected sturgeon showed that the number of gut microbial species decreased in infected sturgeon, the number of some intestinal commensal bacteria decreased, and the balance of the intestinal microorganisms was disrupted. Histopathological sections indicated many inflammatory cells, congestion, and even necrosis in the tissue of diseased sturgeon. Analysis of blood indexes revealed an increase in the proportion of mononuclear cells and a decrease in the proportion of lymphocytes in the peripheral blood of diseased sturgeon. Significantly elevated serum levels of aspartate aminotransferase and alanine aminotransferase, whereas alkaline phosphatase, total protein, albumin, and globulin were decreased in diseased sturgeon. Antimicrobial susceptibility tests demonstrated that D-59 is susceptible to florfenicol, enrofloxacin, and neomycin sulfate. This study aimed to highlight the dangers of *Staphylococcus sciuri* infection during hybrid sturgeon culture and to provide recommendations for diagnosis and treatment.

## Highlight

1. *Staphylococcus sciuri* (D-59) was isolated from hybrid sturgeon and identified as the pathogenic bacteria.
2. The strain was susceptible to florfenicol, enrofloxacin, neomycin sulfate, doxycycline, ampicillin, penicillin, gentamicin, minocycline, tetracyclines, and amikacin.
3. Pathological sections showed significant pathological changes in the liver, spleen, kidney and intestine.
4. *S. sciuri* infection has led to a disruption of intestinal homeostasis and a reduction in the number of intestinal commensal bacteria.
5. Haematological parameters indicators show severe impairment of liver function in affected sturgeon.

## 1 Introduction

*Acipenser sinensis* is a species of Acipenseriformes, Acipenseridae, and *Acipenser*, the evolutionary rate of which is slower than other vertebrates and is known as a living fossil (Bemis et al., 1997). The main species of sturgeon cultivated in China are *Acipenser baerii*, *Acipenser schrenckii*, and hybrid sturgeon (Wei et al., 2004). Sturgeon are giant fish, with late sexual maturity and a long breeding cycle during the culture process; therefore, cross-breeding is an effective way to improve its germplasm. The main commercially farmed hybrid sturgeon species in China include *Huso dauricus*♀ × *Acipenser schrenckii*♂, *Acipenser schrenckii*♀ × *Huso dauricus*♂, and *Acipenser baerii* ♀ × *Acipenser schrenckii* ♂, which have the characteristics of good adaptation, fast growth rate, early sexual maturity (5–6 years old), fertile offspring, and stable genetic traits (Wei et al., 2004). In recent years, the sturgeon aquaculture industry has attracted much attention, and the circulation of sturgeon roe, sturgeon fry, and commercial sturgeon in the markets has risen significantly (Bronzi et al., 2019). Nevertheless, diseases of sturgeon are increasing annually, causing growing amounts of damage. There have been reports of bacterial infection in sturgeon culture in China, such as by *Aeromonas hydrophila* (Meng et al., 2011), *Plesiomonas shigelloides* (Jiang et al., 2021), and *Vibrio metschnikovii* (Xiao et al., 2022).

Widespread mortality of hybrid sturgeon caused by bacterial infection occurred in a sturgeon farm in Yichang, Hubei Province, China, in the summer of 2021.We inquired with the farm owner that initially only two ponds of sturgeon showed slow movement, decreased appetite, severe anal redness and swelling, and surface bleeding. Half a month later, the disease spread to ten adjacent ponds, where the diseased fish were severely inappetent, slow-moving, with red, swollen anuses and bleeding bodies, the mortality rate was increasing day by day, up to about 25% per day. During this period, treatment with antibiotics such as sulfonamide was not effective. We measured the temperature and dissolved oxygen of the water in the affected ponds, the temperature of all ten ponds was stable at 18°C and the dissolved oxygen was sufficient at 6mg/L. We then isolated and tested for parasites, common viruses and bacteria in sturgeon, and did not detect parasites or viruses, but isolated one strain of bacteria. In this study, a strain of bacteria named D-59 was isolated from the diseased sturgeon and identified as *Staphylococcus sciuri* by isolation and purification, Gram staining, microscopic observation, and physiological and biochemical tests. The pathogenicity of strain D-59 was verified by regression infection experiments on healthy hybrid sturgeon. Subsequently, to investigate its pathogenesis, high-throughput sequencing was used to detect microbial changes in the intestinal tract after infection with strain D-59 and to assess hematological and histopathological indicators in the diseased sturgeon. The susceptibility of this pathogenic bacterium to different antibiotics was clarified using antimicrobial susceptibility test. To date, there have been no reports that *S. sciuri* can cause disease in hybrid sturgeon. This study aimed to provide a scientific reference for the diagnosis and treatment of *S. sciuri* infection in hybrid sturgeon.

## 2 Materials and methods

### 2.1 Fish

Diseased sturgeon (*Acipenser baerii* ♀ × *Acipenser schrencki* ♂) were collected from a sturgeon breeding company in Yichang, Hubei Province and the healthy sturgeon were acquired from a sturgeon farm in Jingzhou, Hubei Province, with a body length of 25 ± 2 cm and body weight of 83.73 ± 6.07 g. Healthy sturgeon were temporarily reared in a recirculating water culture system for 14 days before infection, with the water temperature maintained at 20°C and fed a standard diet twice daily. All animal experiments were approved by the Animal Experimental Ethical Inspection of Laboratory Animal Centre, Yangtze River Fisheries Research Institute, Chinese Academy of Fishery Sciences (ID Number: YFI 2021-zhouyong-07).

### 2.2 Sample collection

Three diseased sturgeons with apparent symptoms of the disease and three healthy fish were randomly selected and euthanized in water with 250 mg/L of ethyl 3-aminobenzoate methane sulfonate (MS-222) (Sigma, St. Louis, MO, USA), then taken out and placed on ice with 75% alcohol sprayed on the surface. A 1% heparin-impregnated syringe was used to collect 550 μL of fresh blood *via* caudal lateral vein puncture, a portion of which was used to make blood smears (n = 3 per fish). The remaining blood was placed in Eppendorf tubes overnight at 4°C, centrifuged at 4000 × *g* for 10 minutes, and the resulting serum was stored at −80°C for subsequent serum biochemical index analysis. Liver, spleen, kidney, and intestine were collected and fixed in 4% paraformaldehyde for histopathological analysis. A portion of the intestinal tissue was placed in 1.5 mL sterile Eppendorf tubes, snap-frozen in liquid nitrogen, and stored at −80°C for intestinal microbiological analysis.

### 2.3 Pathogen isolation

Livers and kidneys of diseased fish were sampled in a biosafety cabinet (ESCO, Singapore) using a sterile inoculation loop. Agar plates comprising brain heart infusion medium (BHI; HopeBio, Qingdao, China) were inoculated with the liver and kidney samples and incubated upside down at 28°C for 24 h. The dominant colonies with consistent morphologies were replated on BHI agar plates. Single colonies were picked and inoculated into BHI liquid medium and incubated at 28°C with 200 rpm shaking until the bacterial density reached an OD_600_ = 0.5. A small quantity of bacterial fluid was taken with a sterile inoculation loop, plated, and used for Gram staining. The bacterial solution was stored in glycerol (25% final concentration). The isolated strain was named D-59.

### 2.4 Morphological observation

A small amount of bacterial solution was aspirated, diluted with phosphate‐buffered saline (PBS), plated, dried at room temperature and then stained using a Gram stain kit (Jiancheng, Nanjing, China) (Liu et al., 2020). Staining and morphological characteristics were observed under a light microscope (Olympus, Tokyo, Japan). The morphological appearance of the bacteria was also observed under a scanning electron microscope (Hitachi, Tokyo, Japan).

### 2.5 Biochemical identification

Using the Biolog bacterial identification kit (Biolog, Hayward, CA, USA) the purified culture of strain D-59 and the strain isolated from the regression infection test were inoculated onto IF-A inoculation solution. The inoculum of D-59 was inoculated into GEN III plates (Biolog) at 100 μL per well, incubated in the Biolog fully automated microbial identification system, and identified automatically.

### 2.6 16S rRNA gene analysis

To further identify strain D-59, bacterial DNA was extracted from strain D-59 using a Bacterial Genomic DNA Kit (Tiangen, China), and amplified by PCR. The primers comprised the universal bacterial primers 27F: 5′-AGAGTTTGATCATGGCTCAG-3′ and 1492R: 5′-TACGGTTACCTTGTTACGACTT-3′ (Loy et al., 2002). Thermal cycling included denaturation at 95 °C for 5 min,the amplification conditions were 35 cycles of 94 °C for 30 s, annealing at 55 °C for 30 s, and extension at 72 °C for 90 s; with a final extension at 72 °C for 10 min (Pei et al., 2021). The amplification products were electrophoresed through a 1.5% agarose gel, isolated, and sent to Wuhan Tianyi Huayu Gene Technology Company Limited (Wuhan, China) for sequencing. The sequencing results were compared with sequences deposited at NCBI (https://www.ncbi.nlm.nih.gov). After retrieving highly similar sequences, multiple alignments were performed using MEGA 7.0 software (Kumar et al., 2016). The neighbor-joining method was to construct phylogenetic trees. 1000 replicate bootstrap analysis was used to estimate the reliability of each tree topology.

### 2.7 Intestinal microbial diversity

Gut samples were taken from healthy and diseased hybrid sturgeon, and bacterial genomic DNA was extracted using a bacterial DNA kit (Omega Biotek, Winooski, VT, USA). The extracted DNA was used as a template for the amplification of the V3–V4 high variable region of the 16S rRNA gene using the GeneAmp 9700 system (Applied Biosystems, Foster City, CA, USA). The primers were 341F 5′-ACTCCTACGGGAGGCAGCAG-3′and 806R 5′-GGACTACHVGGGTWTCTAAT-3′ (Xu et al., 2022).Thermal cycling comprised an initial denaturation at 95°C for 2 min, the amplification conditions were 30 cycles of 95°C for 15 s, annealing at 55°C for 30 s, and extension at 72°C for 30 s; with a final extension at 72°C for 10 s. After checking the DNA quality using 1% agarose gel electrophoresis, the samples were sequenced on the Illumina MiSeq PE300 high-throughput sequencing platform (Illumina Inc., San Diego, CA, USA). The composition of the gut microbiota was analyzed at the phylum and genus levels in the healthy and diseased groups using the R software. Alpha-diversity indicators and beta-diversity indicators of the gut microbiota were calculated at the website (http://www.genescloud.cn/analysisProcess). Principal coordinates analysis (PCoA) was used to analyze the operation taxonomic units (OTUs) for each sample.

### 2.8 Pathogenicity

Infection tests for pathogenicity were performed using bacteria isolated from diseased fish tissues. The purified D-59 strain was incubated in BHI liquid medium for 20 h at 28°C with shaking at 200 rpm.Using the plate colony counting method to measure the concentration of bacterial solution, take 1 ml of bacterial solution to make 10 different 10-fold incremental dilutions, then take out 100 μl from each dilution and spread it evenly on BHI agar medium, 28°C for 24 h. Record the number of colonies formed in each dish, and calculate the total number of live bacteria per mL of the original sample based on the dilution times. The remaining bacterial solution was put at 4°C. After the concentration of the bacterial solution is calculated, centrifuged at 4000 *× g* for 10 min. The supernatant was discarded and the pellet was resuspend in PBS to obtain dilutions of 1×10^5^CFU/mL, 1×10^6^CFU/mL, 1×10^7^CFU/mL, 1×10^8^CFU/mL, 1×10^9^ CFU/mL. Then, 180 healthy sturgeon were temporarily kept in the tank for 14 days and randomly divided into 6 groups of 30 fish each, including 5 experimental groups and 1 control group. After anesthesia using MS-222, 0.3 mL of sterile PBS was injected intraperitoneally in the control group, and 0.3 mL of different concentrations of bacteria were injected intraperitoneally in the test groups, respectively. Mortality was counted daily, and dying fish with obvious signs of morbidity were collected for aseptic dissection to re-isolate the bacteria. The Reed‐Muench method was used to calculate the median lethal dose (LD_50_) of strain D‐59 toward hybrid sturgeon (Reed, 1938).

### 2.9 Hematological analysis

#### 2.9.1 Differential white blood cell count

Monocytes, eosinophils, neutrophils, and lymphocytes were sorted and counted in the peripheral blood of diseased sturgeon. Air-dried blood smears were stained with Richter-Gimza staining solution (Chen et al., 2019). Leukocytes were counted under a light microscope with 200 leukocytes per blood smear for lymphocytes, granulocytes, and monocytes (Martins et al., 2009).

#### 2.9.2 Serum biochemical indicators

Measurement of serum biochemical parameters, including alanine aminotransferase (ALT), aspartate aminotransferase (AST), alkaline phosphatase (AKP); and blood protein metabolism parameters, globulin (GLB), total protein (TP) and albumin (ALB) was accomplished using a fully automated biochemical analyzer (Sysmex, Kobe, Japan) (Bayunova et al., 2002).

### 2.10 Histopathological observation

Tissue samples were fixed with 4% paraformaldehyde for 24 h. Dehydrated in 70%, 80%, 90%, 95%, and 100% ethanol, permeabilized with xylene twice,waxed twice(for about 35 min each time). The samples were embedded in paraffin, sectioned (at about 5 μm), stained with hematoxylin-eosin (HE), and observed under a light microscope (Olympus) to determine the pathological changes of the tissue.

### 2.11 Antimicrobial susceptibility test

Antimicrobial susceptibility testing was performed according to CLSI standards (CLSI, 2021). CAMHB was used to determine the MIC of florfenicol, enrofloxacin, neomycin sulfate, doxycycline, ampicillin, penicillin, gentamicin, minocycline, sulfanilamide, tetracyclines, amikacin, polymyxin B drug-sensitive paper sheets were purchased from Hangwei (Hangwei, Hangzhou, China). D-59 bacterial solution (100 μL) was spread evenly on a CAMHB agarose plate using a sterile applicator stick. After the medium was dried, drug-sensitive paper sheets were placed on the agar surface and the plates were incubated at 28°C for 24 h. The diameter of the inhibition circle around each drug‐sensitive paper sheet was measured using vernier calipers (Xia et al., 2019). The susceptibility of the strains to the antibiotics was evaluated by the diameter of the bacterial inhibition circle with reference to the drug sensitive paper sheet instructions (CLSI, 2018).

### 2.12 Statistics

The data were analyzed using SPSS software version 19.0 (IBM Corp., Armonk, NY, USA). Statistical analysis was performed using analysis of variance (ANOVA). The significance of differences between means was evaluated using Duncan’s multiple range test. Differences were considered significant at a level of *P* < 0.05.

## 3 Results

### 3.1 Clinical symptoms

**Figure 1:**
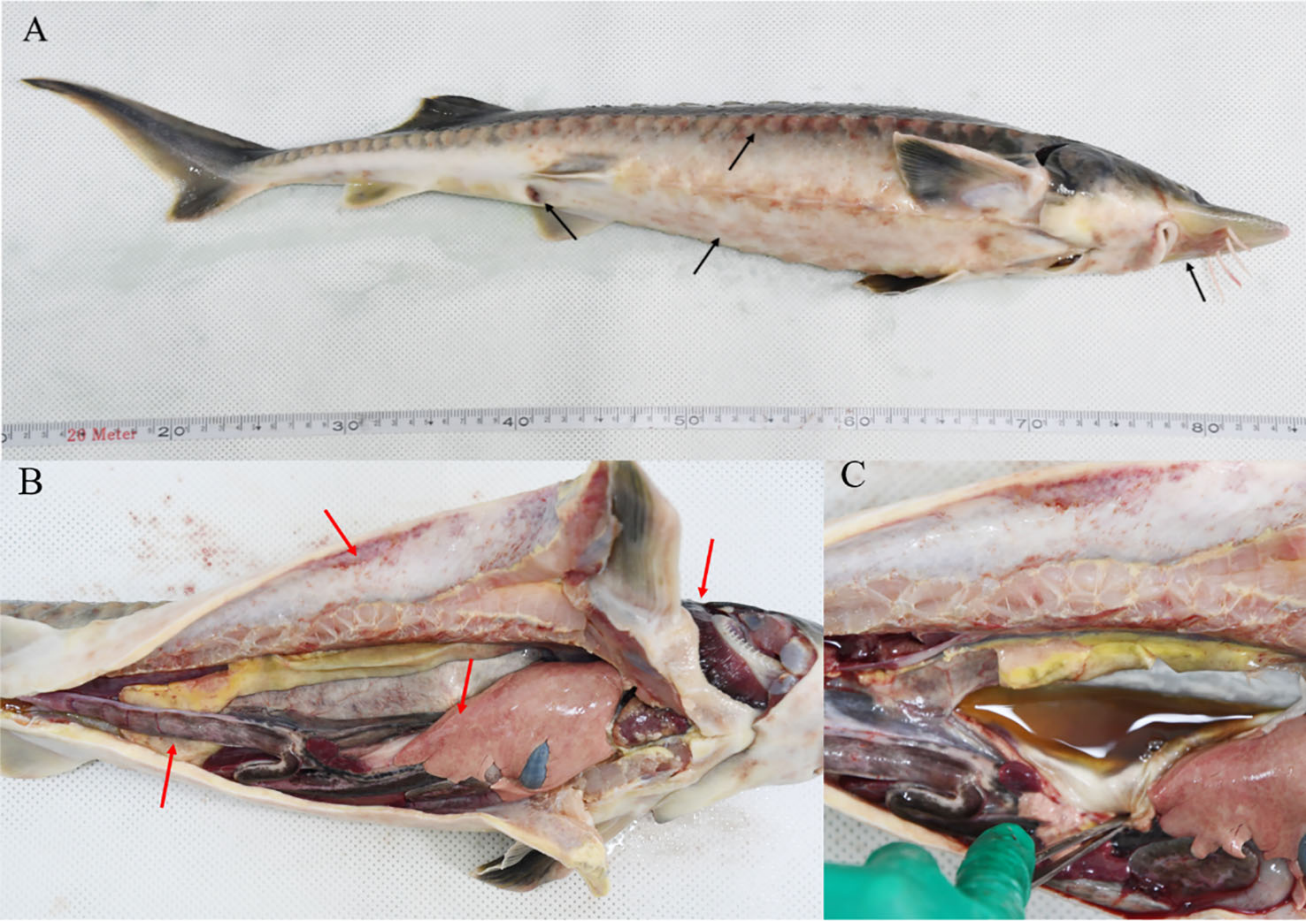
Clinical symptoms of hybrid sturgeon: **(A)** Bleeding around the mouth, abdomen, and both sides of the bony plate; redness and swelling of the anus; whitening of the ventral fin with slight ulceration (black arrows). **(B)** Bleeding gill filaments, bleeding muscles, swollen liver with bleeding spots, red-black intestinal congestion (red arrows). **(C)** Ascites.

Bleeding around the mouth, abdomen, and two bone plates, a red and swollen anus, part of the pelvic fin was white, and mild ulceration were observed (**Figure 1A**). An autopsy showed that the diseased fish had congested gill filaments, bleeding muscles, enlarged liver with bleeding spots, congested red-black intestines (**Figure 1B**), and hemorrhagic ascites (**Figure 1C**). The microscopic examination did not reveal parasitic worms or fungal infections on the body surface, gill filaments, or fins of the diseased fish.The tissues of the diseased sturgeon were subsequently tested for white sturgeon iridovirus (WSIV), white sturgeon herpesviruses-I, II (WSHV-I, II), and Shovenose sturgeon iridovirus (SSIV), and the PCR results were negative.

### 3.2 Morphological characteristics

**Figure 2:**
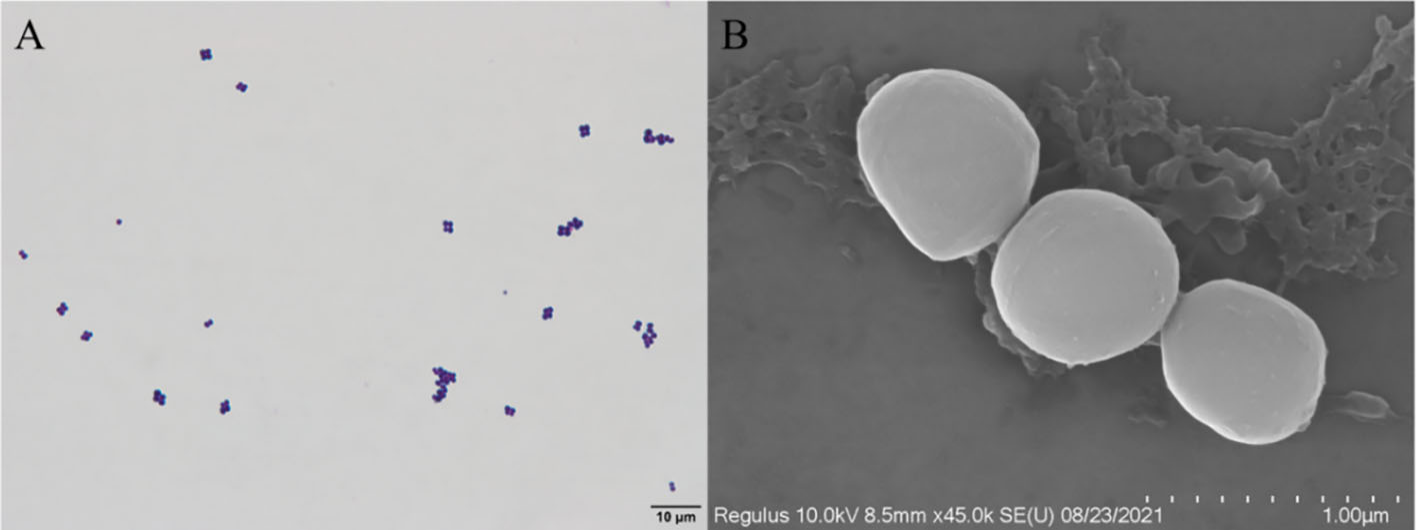
Morphological characteristics of strain D-59: **(A)** Gram stain (scale bar 10 μm); **(B)** Scanning electron microscopy (scale bar 1.00 μm).

The isolated D-59 strain was incubated on BHI plates at 28°C for 24 h, forming white or creamy white round colonies with a diameter of 1–2 mm, with smooth surfaces, neat, rounded, and raised edges. Gram staining, and light microscopy revealed cells arranged as spherical grape bunches or individually scattered gram-positive cocci (**Figure 2A**). Scanning electron microscopy allowed observation of spherical-shaped organisms with a diameter of approximately 0.50 μm (**Figure 2B**).

### 3.3 Biochemical identification

Biochemical tests were carried out on isolated bacteria using a biochemical analyzer, and the results are shown in **Table 1**. D-59 was positive for Dextrin, D-maltose, D-Trehalose, D-cellobiose, Gentiobiose, Sucrose, D-Turanose, Stachyose, and acidic pH 6 reaction. The reactions were negative for lincomycin, niaproof, mucic Acid, quinic acid, vancomycin, tetrazolium blue, p-hydroxy-phenylacetic acid, D-malic acid, and L-fucose. Based on the above morphological observations and biochemical identification results, the isolate was tentatively identified as *Staphylococcus sciuri*.

**Table 1:** Physiological and biochemical identification of D-59.

	Reaction item	Result*		Reaction item	Result*
A1	Negative control	N	E1	Gelatin	P
A2	Dextrin	P	E2	Glycyl-L-Proline	B
A3	D-Maltose	P	E3	L-Alanine	P
A4	D-Trehalose	P	E4	L-Arginine	B
A5	D-Cellobiose	P	E5	L-Aspartic Acid	B
A6	Gentiobiose	P	E6	L-Glutamic Acid	P
A7	Sucrose	P	E7	L-Histidine	B
A8	D-Turanose	P	E8	L-Pyroglutamic Acid	B
A9	Stachyose	B	E9	L-Serine	B
A10	Positive control	P	E10	Lincomycin	N
A11	Acidic PH PH6	P	E11	Guanidine HCl	B
A12	Acidic PH PH5	N	E12	Niaproof 4	N
B1	D-Raffinose	B	F1	Pectin	P
B2	α-D-Lactose	B	F2	D-Galacturonic Acid	B
B3	D-Melibiose	B	F3	L-Galactonic Acid Lactone	B
B4	β-Methyl-D-Glucoside	P	F4	D-Galactonic Acid	P
B5	D-Salicin	P	F5	D-Glucuronic Acid	P
B6	N-Acetyl-D-Glucosamine	P	F6	Glucuronamide	P
B7	N-Acetyl-β-Maanosamine	P	F7	Mucic Acid	N
B8	N-Acetyl-D-Galactosamine	P	F8	Quinic Acid	N
B9	N-Acetyl Neuraminic	B	F9	D-Saccharic Acid	B
B10	1% NaCl	P	F10	Vancomycin	N
B11	4% NaCl	P	F11	Tetrazolium Violet	B
B12	8% NaCl	P	F12	Tetrazolium Blue	N
C1	α-D-Glucose	P	G1	P-Hydroxy-Phenylacetic Acid	N
C2	D-Mannose	P	G2	Methyl Pyruvate	B
C3	D-Fuctose	P	G3	D-Lactic Acid Methyl Ester	B
C4	D-Galactose	P	G4	Lactic Acid	P
C5	3-Methyl Glucose	B	G5	Citric Acid	B
C6	D-Ducose	B	G6	α-Keto-Glutaric Acid	P
C7	L-Fucose	N	G7	D-Malic Acid	N
C8	L-Rhamnose	B	G8	L-Malic Acid	P
C9	Inosine	B	G9	Bromo-Succine-Acid	B
C10	1% Sodium Lactate	B	G10	Nalidixic Acid	B
C11	Fusidic Acid	N	G11	Lithium Chloride	P
C12	D-Serine	B	G12	Potassium Tellurite	B
D1	D-Sorbitol	P	H1	Tweem 40	P
D2	D-Mannitol	P	H2	γ-Amino-Butyric-Acid	P
D3	D-Arabitol	P	H3	α-Hydroxy- Butyric-Acid	P
D4	Myo-lnositol	P	H4	β–Hydroxy-D, LButyric-Acid	B
D5	Glycerol	B	H5	α-Keto-Butyric Acid	B
D6	D-Glucose-6-po4	P	H6	Acetoacetic Acid	P
D7	D-Fructose-6-po4	P	H7	Propionic Acid	B
D8	D-Aspartic acid	B	H8	Acetic Acid	P
D9	D-Serine	N	H9	Formic Acid	B
D10	Troleandomycin	N	H10	Aztreonam	P
D11	Rifamycin SV	N	H11	Sodium Butyrate	B
D12	Minocycline	N	H12	Sodium Bromate	N

*P, positive; N, negative; B, borderline; L, less than the A1 well.

### 3.4 16S rRNA gene sequence analysis

**Figure 3:**
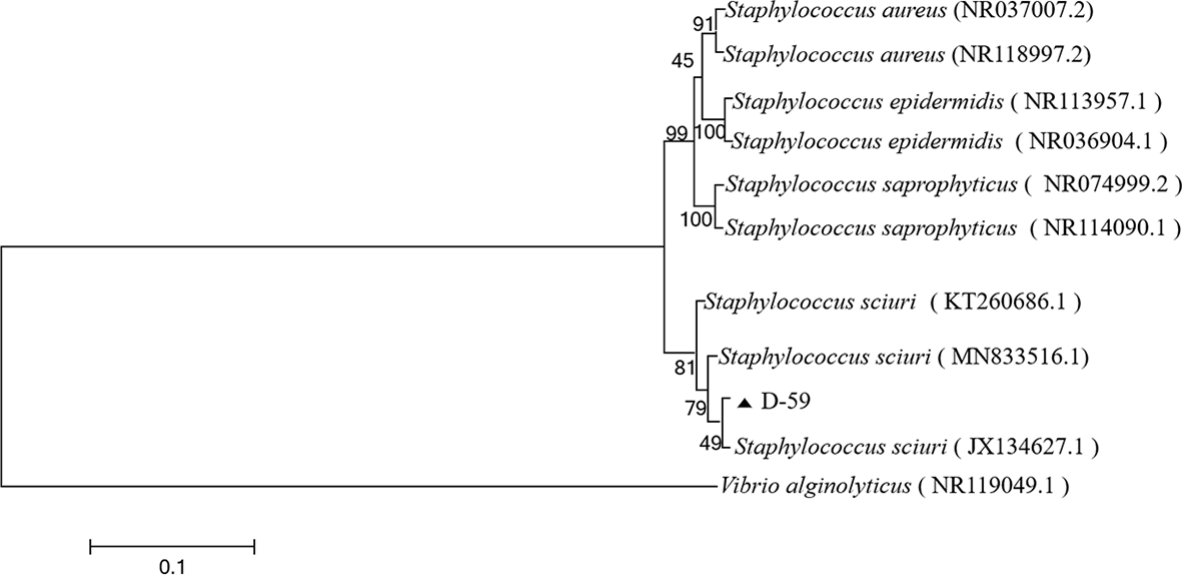
Phylogenetic tree of strain D-59. The phylogenetic relationships of the D-59 genotype identified in this study with the 16S rRNA nucleotide sequences of other reported genotypes. The MEGA 7.0 program was used to construct the phylogenetic tree using multiple sequences matching the sequences of nine members of the genus *Staphylococcus* and the Neighbor-joining method. The number at each branch indicates the percentage of bootstrap values for 1000 replicates. The scale bar indicates the number of substitutions per site.

The sequencing results were analyzed by BLAST in the NCBI GenBank database, and the sequences with high similarity were found to be the 16S rRNA gene sequences of *S. sciuri* strains (**Figure 3**). The BLAST search results indicated that the 16S rRNA gene fragment of isolate D-59 (SRR20887062) clustered with *S. sciuri* (JX134627.1).These results proved that the bacterium was *S. sciuri* at the molecular level.

### 3.5 Intestinal microorganisms

**Figure 4:**
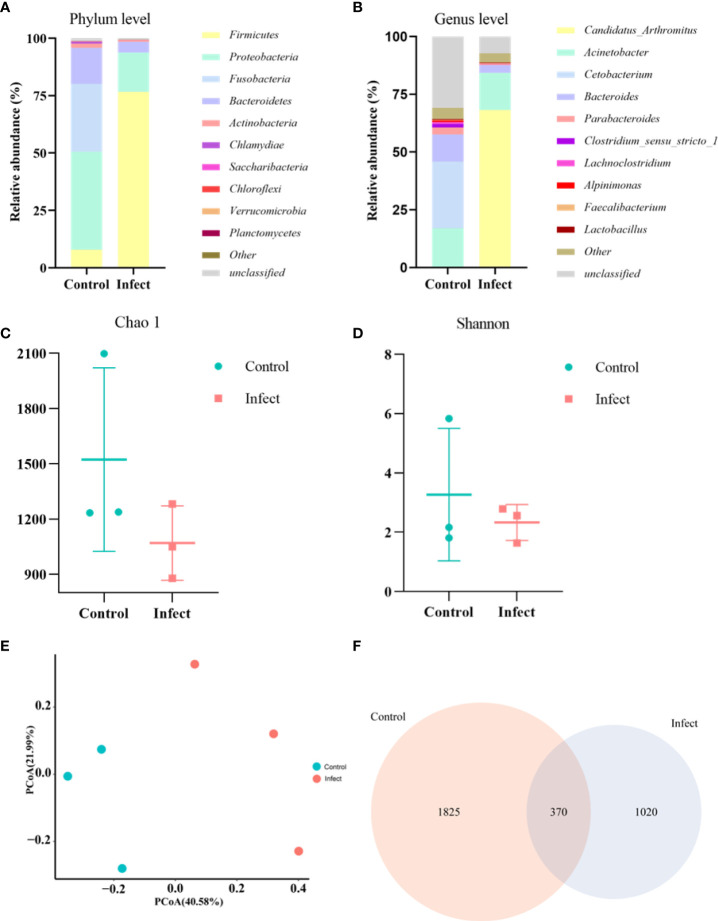
Gut microbial analysis. **(A)** Relative abundance at the phylum level. **(B)** Relative abundance at the genus level. **(C)** Chao1 index. **(D)** Shannon index. **(E)** Unweighted unifrac PCoA. **(F)** A Venn diagram showing the number of unique and shared operational taxonomic units (OTUs) of gut bacteria of healthy sturgeon and D-59 infected sturgeon.

To study the microbiota in the gut of sturgeon infected with *S. sciuri*, we performed 16S rRNA gene sequencing. The phylum level bacterial taxonomic composition of the gut of healthy sturgeon showed a high abundance of *proteobacteria* (43%), followed by *fusobacteria* (30%), *bacteroidetes* (16%), and *firmicutes* (8%). The abundance of some microflora in the gut of diseased sturgeon was altered. The abundances of *proteobacteria* (17%), *Fusobacteria* (0.1%), and *Bacteroidetes* (4.5%) were reduced in the diseased group compared with those in the healthy group. However, the abundance of *firmicutes* (76%) was increased (**Figure 4A**). Higher abundance at the genus level was observed for *Cetobacterium* (29%), *Acinetobacter* (17%), *Bacteroides* (11%), and *Parabacteroides* (3%) (**Figure 4B**). The diseased group had an elevated abundance of *Candidatus_Arthromitus* (68%). The numbers of *Cetobacterium* (0.08%), *Bacteroides* (3.3%), and *Parabacteroides* (0.3%) were decreased (**Figure 4B**). Analysis of the chao1 and Shannon indices showed a significant decrease in the chao1 index and no significant change in the Shannon indices in the diseased group (**Figures 4C, D**). This indicated that *S. sciuri* infection led to a reduction in the number of microbial species in the gut of sturgeon, but did not lead to changes in microfloral diversity. Principal coordinate analysis (PCoA) plots based on unweighted Unifrac metrics were used to detect flora relationships in the healthy and diseased groups (**Figure 4E**). The results showed that the distributions of intestinal microorganisms in the normal and diseased groups were in different regions. This suggested that the invasion of *S. sciuri* altered the composition of the intestinal microorganisms. To understand the differences in OTUs between the diseased group and the healthy group, all OTUs with an average abundance greater than 1 were selected for Venn diagram analysis (high-abundance union OTUs). A total of 370 OTUs were shared by all samples, whereas the numbers of unique OTUs were1825 in the group control group, 1020 in the D-59 group (**Figure 4F**).

### 3.6 Pathogenicity

**Figure 5:**
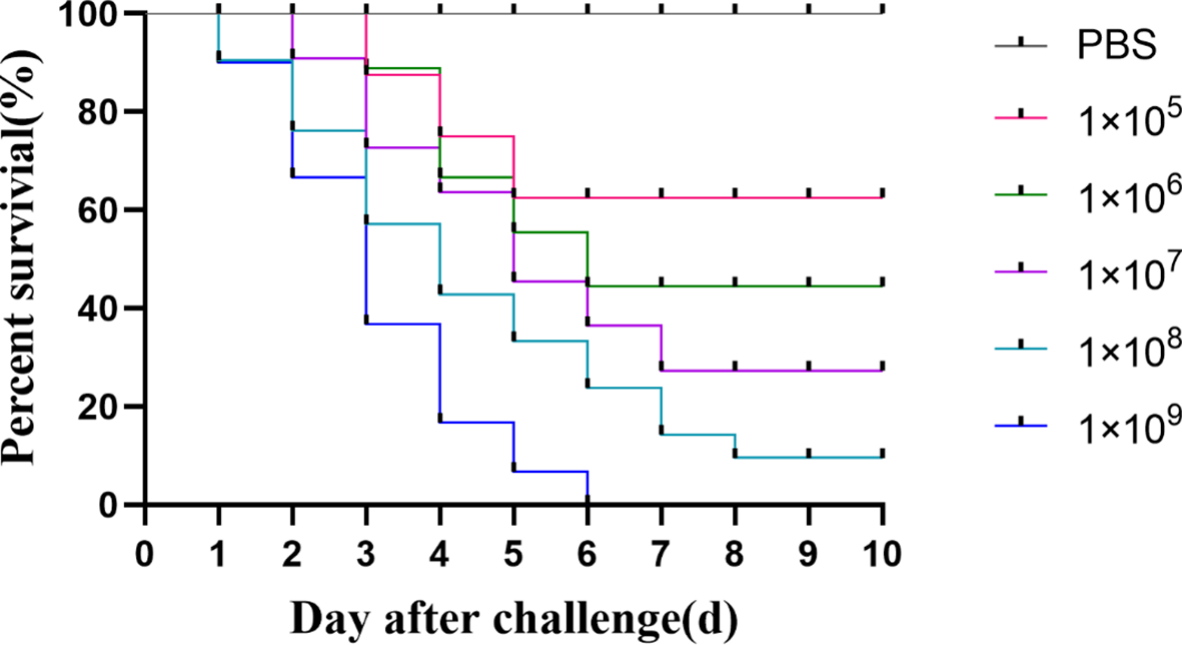
Regression infection analysis of D-59. Survival of hybrid sturgeon by injection of different concentrations of D-59 strain: The control group was injected intraperitoneally with 0.3 mL of sterile PBS, and the test group was injected intraperitoneally with 0.3 mL of 1 × 10^5^ CFU/mL,1 × 10^6^ CFU/mL,1 × 10^7^CFU/mL,1 × 10^8^ CFU/mL, 1 × 10^9^ CFU/mL bacterial fluids, and was monitored for 10 d.

As shown in **Figure 5**, sturgeon in the group injected with the bacterial solution concentration of 1×10^5^ CFU/mL began to die on day 3, and the survival rate was 70% after 5 days of inoculation and remained unchanged. However, sturgeon inoculated with 1 × 10^9^ CFU/mL showed mortality from the first day, reaching 100% at 6 days after inoculation. The LD_50_ of strain D-59 was calculated as 2.60 ± 0.19 × 10^3^CFU/g. Sequencing of 16S rRNA gene of the bacteria isolated from sturgeon with obvious signs of disease were compared with those of *S. sciuri*.

### 3.7 Differential white blood cell count

**Figure 6:**
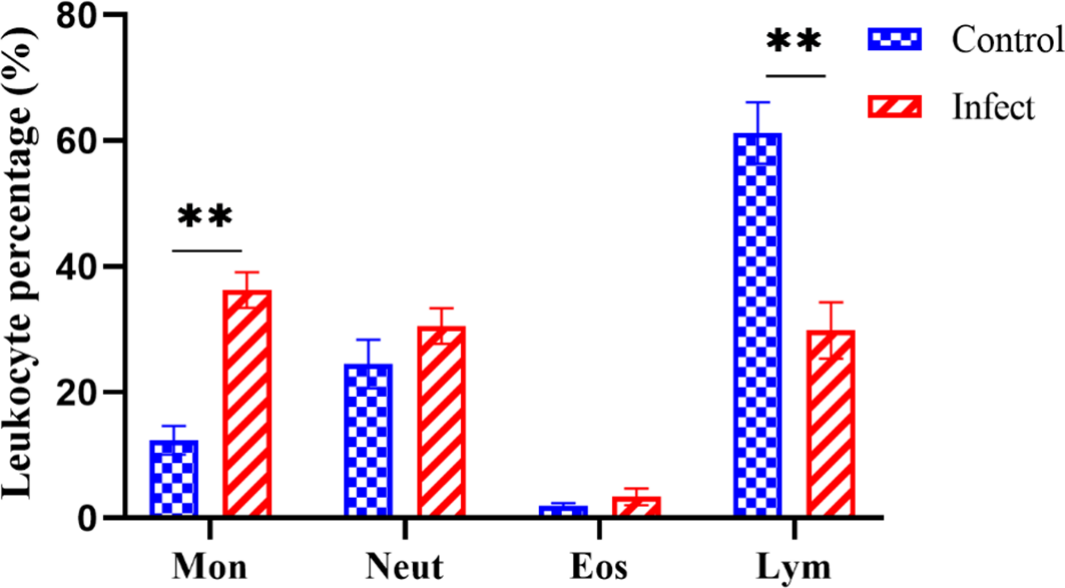
Differential white blood cell count. Mon: monocytes; Neut: neutrophils; Eos: eosinophils; Lym: lymphocytes; the proportion of monocytes was significantly increased (*P* < 0.01) and the proportion of lymphocytes was significantly decreased (*P* < 0.01) in diseased sturgeons. Although there was a trend of increase in neutrophils and eosinophils, it was not statistically significant (*P* > 0.05). **p < 0.01.

As shown in **Figure 6**, D-59 infection of hybrid sturgeon affected its blood parameters. Leukocyte sorting counts were performed by making blood smears from the peripheral blood of healthy and diseased sturgeon. The number of monocytes accounted for 12.32% of leukocytes in the control group, but was significantly higher in the bacterially infected group, reaching 36.69% (*P* < 0.01).The number of lymphocytes in the blood was significantly lower in the diseased group (30.16%) compared to the healthy group (61.01%) (*P* < 0.01). Although there was a trend towards an increase in neutrophils and eosinophils, it was not statistically significant (*P* > 0.05).

### 3.8 Serum biochemical indicators

**Figure 7:**
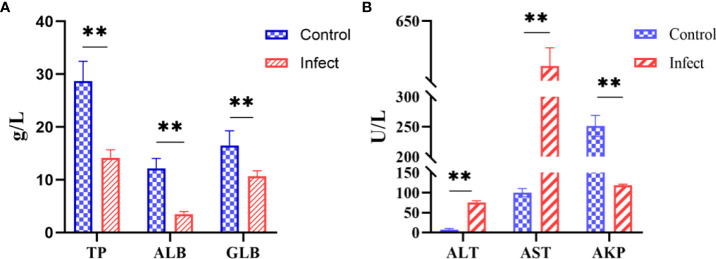
Serum biochemical indices. **(A)** Significant increase in alanine aminotransferase (ALT), aspartate aminotransferase (AST), alkaline phosphatase (AKP) (*P* < 0.01). **(B)** Significant decrease in total protein (TP), albumin (ALB), and globulin (GLB) in the serum of sturgeon infected with D-59 (*P* < 0.01). **P < 0.01.

The serum biochemical parameters of diseased and healthy sturgeons were compared. Compared with those in the healthy fish, the alanine aminotransferase level increased from 7.7 U/L to 75.3 U/L and the aspartate aminotransferase level increased from 100 U/L to 575 U/L (**Figure 7A**, *P* < 0.01); the level of alkaline phosphatase decreased from 251.7 U/L to 118.3 U/L (**Figure 7A**, *P* < 0.01). Serum total protein (14.2 g/L), albumin (3.5 g/L), and globulin (10.7 g/L) were significantly lower (**Figure 7B**, *P* < 0.01) in sturgeons infected with D-59 compared with those in healthy fish (total protein (28.7 g/L), albumin (12.1 g/L) and globulin (16.6 g/L) (**Figure 7B**).

### 3.9 Histopathological observation

**Figure 8:**
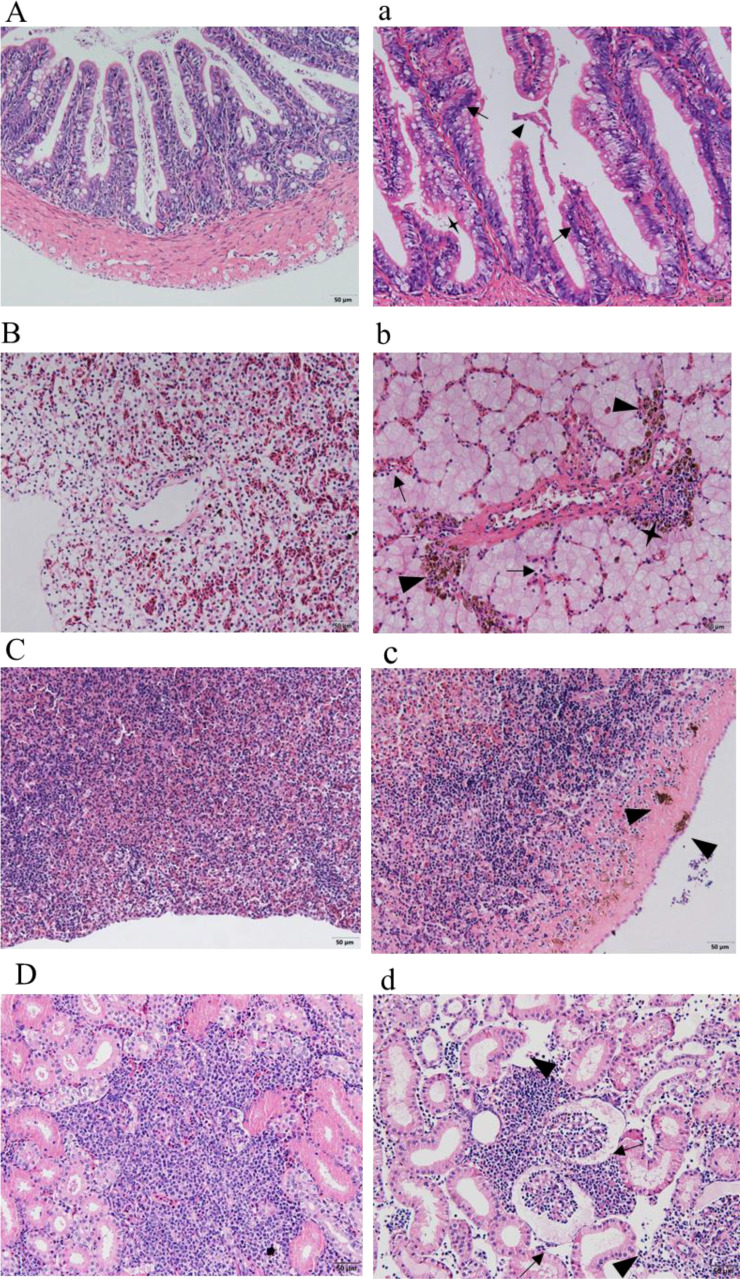
Histopathological observation. **(A–D)** show the intestines, livers, spleens, and kidneys of healthy sturgeons, respectively. **(a)** Intestinal pathological changes; intestinal epithelial cell hyperplasia (black arrows), shedding of the intestinal mucous membrane (triangles), necrotic (quadrangular). **(b)** Changes in liver pathology; dilated hepatic sinusoids (black arrows), infiltrated inflammatory cells (quadrangular), increased number of perivascular melanocyte macrophage centers (triangles). **(c)** Pathological changes in the spleen; increased numbers of melanocyte macrophage centers (triangles). **(d)** Kidney pathological changes; swollen renal glomeruli (black arrows), necrosis or even disappearance of interstitial kidney tissue (black arrows). Scale bar: 50 μm, magnification 20× objective lens.

Sections of intestinal, liver, spleen, and kidney tissues from healthy sturgeon are shown in (**Figures 8A–D**), and tissue sections of the intestine, liver, spleen and kidney of the diseased sturgeon are shown in (**Figures 8a–d**). Apparent lesions were observed in the diseased liver, spleen, kidney, and intestine tissues of the diseased fish. The intestinal villi were disorganized, and intestinal epithelial cell hyperplasia and shedding of the necrotic intestinal mucous membrane were observed (**Figure 8a**). Dilated hepatic sinusoids and perivascular inflammatory cell infiltration were observed in the diseased group but not in the healthy group (**Figure 8b**). A significant increase in melanocyte macrophage centers was observed in the liver and spleen of the infected group (**Figures 8b, c**). There was significant pathology in the kidneys of the infected group, such as swollen renal glomeruli, and necrosis or even disappearance of interstitial kidney tissue (**Figure 8d**).

### 3.10 Antimicrobial susceptibility test

Antimicrobial susceptibility test were performed on D-59 using a variety of antimicrobial analogs. The susceptibility of D-59 to each drug was calculated based on the diameter of the inhibition circle. D-59 was observed to be sensitive to florfenicol, enrofloxacin, neomycin sulfate, doxycycline, ampicillin, penicillin, gentamicin, minocycline, tetracyclines, and amikacin. In contrast, D-59 was moderately sensitive to polymyxin B and resistant to sulfonamide (**Table 2**).

**Table 2:** D-59 Antimicrobial susceptibility test.

Medicine name	Concentration	Inhibition Zone (mm)	Sensitivity*
Florfenicol	30 μg	17	S
Enrofloxacin	10 μg	28	S
Neomycin sulfate	30 μg	18	S
Doxycycline	30 μg	28	S
Ampicillin	10 μg	27	S
Penicillin	10 U	32	S
Gentamicin	10 μg	23	S
Minocycline	30 μg	30	S
Sulfanilamide	30 μg	6	R
Tetracyclines	30 μg	28	S
Amikacin	30 μg	22	S
Polymyxin B	300 IU	13	I

*S, susceptible; I, intermediate; R, resistant.

## 4 Discussion

The gram-positive cocci, *S. sciuri*, is a taxonomically primitive species of *Staphylococci*, which is widely present in nature and is associated with many farm animals and their food, pet animals, and various wildlife (Aldridge, 1988; Piessens et al., 2012). Zoonotic transfer of *Staphylococcus* is common between domestic animals and humans, and can be transmitted through direct contact, environmental contact, and animal-derived food processing (Mama et al., 2019). In addition, dust and surface water can also serve as vectors of transmission of this bacterium (Couto et al., 2000; Dakic et al., 2005; Gomez et al., 2017). *S. sciuri* has high salt tolerance (up to 27%) and a wide survival temperature range (4°C– 45°C), and is thus well-adapted to extreme ecological conditions (Majumdar and Gupta, 2020). In the case of staphylococcal infections, mechanical barriers such as skin and mucous membranes are not sufficient to provide protection to the host, as oral ingestion or skin contact are the main routes of infection (Dakic et al., 2005). Some components of *Staphylococcus* might suppress the innate immune response in the early stages of *Staphylococcus* infection, and previous studies found that *S. sciuri* infection of neonatal mice suppressed macrophage function in the early stages of infection, thereby promoting bacterial colonization of tissues (Chen et al., 2007; Li et al., 2011). *S. sciuri* suppresses innate immune factors in corals, leading to widespread coral disease (Divya et al., 2018). Canak and Timur first isolated *S. sciuri* from a mixed bacterial infection of *Sparus aurata* (Canak and Timur, 2020). Wang et al. reported the isolation of *S. sciuri* in yellow catfish (*Pelteobagrus fulvidraco*) that died of bacterial disease (Wang et al., 2017). In the present study, highly pathogenic *S. sciuri* was isolated from cultured hybrid sturgeon. The isolated bacteria were identified by Gram staining combined with 16S rRNA gene sequence analysis. The regression infection experiment revealed that the disease was consistent with that of natural-onset sturgeon, the LD_50_ was calculated as 2.60 ± 0.19 × 10^3^ CFU/g. *S. sciuri* can be isolated from artificially infected sturgeon in accordance with *Koch’s* law and is thus one of the pathogenic bacteria that can cause disease in hybrid sturgeon.

As the largest immune organ in large fish, the intestinal microbiota is essential to regulate the immune system of aquatic animals and prevent the invasion of pathogenic microorganisms (Ma et al., 2018; Filipp et al., 2019). Maintaining a dynamic balance and stability of the microbiota in the intestinal environment is crucial to maintain the host’s health; under unfavorable conditions, the balance of the host intestinal microbiota is disturbed (Blander et al., 2017). Significant changes in intestinal microorganisms is a potential diagnostic criterion for disease (Shin et al., 2015). In this study, the abundance of the *Firmicutes* and *Candidatus_Arthromitus* reached 77% and 68%, respectively, at the phylum level in the diseased group, and the abundances of *Proteobacteria*, *Fusobacteria*, *Bacteroidetes*, *Cetobacterium*, *Bacteroides*, *Parabacteroides* groups decreased. Although *Firmicutes* has been considered as a beneficial bacterium among intestinal microorganisms, the abnormal increase in *Firmicutes* and the decreased abundance of *Bacteroidetes* in the diseased group might be caused by obesity, as evidenced by the hepatocyte-filled adipocytes in the pathological sections of the liver diseased group. Increased abundance of *Candidatus_Arthromitus* is considered as a possible cause of enterocolitis (Del-Pozo et al., 2010; Wang et al., 2021). Reduced abundance of colonized probiotic bacteria in the gut weakens the intestinal epithelial barrier and can cause failure of transmission of signals to the host that regulate the immune system (Bermudez-Brito et al., 2012). Sturgeon infected with *S. sciuri* have a reduced abundance of colonized commensal bacteria in the gut, and the inability of probiotic bacteria to communicate with the host through pattern recognition receptors would lead to enteritis. A similar decrease in abundance was observed in grass carp with enteritis (Tran et al., 2018). Overall, during *S. sciuri* infection, the reduction in gut microbial species intestinal colonizing commensal bacteria decreased led to the development of enteritis.

ALT, AST, and AKP are important enzymes to assess fish health, and their levels are related to liver tissue damage, various diseases, parasitic infections, and poisoning (Xu et al., 2021). Elevated serum levels of ALT and AST might reflect the transfer of enzymes to the serum caused by liver damage (Shahsavani et al., 2010). This corresponds to the symptoms of severe hemorrhage in the liver of the diseased sturgeon. The innate immunity parameter AKP is commonly used to monitor non-specific immunity in fish. AKP acts as an important lysosomal enzyme in fish, hydrolyzing and digesting invading pathogens during the immune response (He et al., 2017). In this study, AKP levels in diseased sturgeon were significantly decreased, suggesting that D-59 infection reduced the non-specific immune defense of sturgeon against pathogenic bacterial invasion. The same serological changes were found in hybrid sturgeon infected with *Vibrio metschnikovii* (Xiao et al., 2022).

Total protein (TP) in serum is synthesized by the liver and includes albumin (ALB) and globulin (GLB) (Sepulveda et al., 2012). Significant reduction in TP, ALB, and GLB were observed in studies of *Streptococcus lactis*-free infected *tilapia*, and *Vibrio harveyi* infected sea bass (Mao et al., 2020; Zhou et al., 2020). Herein, similar changes were observed in the serum proteins of hybrid sturgeon after D-59 infection, which might be closely related to the effect of *S. sciuri* infection on protein synthesis in the liver.

Usually the number of white blood cells (WBCs) in peripheral blood can be used as an indicator to determine the presence of infectious diseases, because healthy fish have fewer WBCs (Shameena et al., 2021a). In the present study, the proportion of monocytes increased significantly in diseased sturgeon. In contrast, the proportion of lymphocytes decreased significantly, which was consistent with the change in WBCs after *Yersinia ruckeri* infection in hybrid sturgeon (Fajardo et al., 2022). The increase in monocytes and neutrophils can be considered a positive response of the host cellular immune system to D-59 infection (Sebastião et al., 2011). In addition, increased WBCs might play an important role during D-59 infection by stimulating the inflammatory response and hematopoietic tissue, thus enhancing the immune system by producing antibodies that can act on the disease (Shameena et al., 2021b). In contrast, the significant decrease in lymphocytes might be related to damage to the liver and spleen tissues and continuous blood loss.

Histopathology is an important method to reveal the pathogenesis of lesions and to make pathological judgements. The histopathology of diseased sturgeon is primarily inflammatory, with some tissue necrosis. Similar histopathological changes were described in sturgeon infected with *Plesiomonas shigelloides* (Jiang et al., 2021), *Streptococcus inia*e (Behera et al., 2018), *Aeromonas hydrophila*, and *Aeromonas veronii* (Di et al., 2018). It is highly likely that this tissue damage is a result of the bacterial toxins produced by *S. sciuri*, which are extremely toxic to organs.

The targeting of *S. sciuri* through drug sensitivity tests, accurate drug administration, precise drug administration, and avoidance of antibiotic abuse is required. D-59 is sensitive to florfenicol, enrofloxacin, neomycin sulfate, doxycycline, ampicillin, penicillin, gentamicin, minocycline, tetracyclines, and amikacin. In contrast, D-59 was moderately sensitive to polymyxin B and resistant to sulfonamide. This differs from the antimicrobial drug susceptibility of 158 strains of *S. sciuri* isolated within the livestock environment, in which more than half of the strains had a high rate of resistance to tetracycline, penicillin, benzocillin, and fusidic acid (Schoenfelder et al., 2016). This suggests that there are differences in drug susceptibility of the same species of bacteria originating from different animal sources or regions. Hence, to avoid drug abuse, antimicrobial drugs should be selected to treat bacterial diseases in fish based on the results of drug sensitivity tests.

*S. sciuri* has poor host specificity and has been isolated from human skin, mastitis in cattle, diseased goats, dogs, rice, aquatic animals, and the aquatic environment (Rahman et al., 2005; Abraham et al., 2017; Abdelsalam et al., 2021). This suggests that its ability to spread among animals, plants, and the environment should be taken seriously. Members of the *S. sciuri* group carry a wide variety of antimicrobial genes, e.g., virulence genes, most of which are also present in other *Staphylococci*. This indicates that *S. sciuri* can serve as a large exchangeable gene pool for other *Staphylococci* and bacterial genera. The use of antibiotics against bacterial diseases is still the primary method used during aquaculture production; therefore, rotating the use of effective antimicrobial drugs to avoid causing the emergence of superbugs would be a feasible approach in in response to diseases caused by *S. sciuri*. In particular, emphasis should be placed on daily management, such as disinfection of utensils during breeding to avoid cross-contamination and prevention of the exchange of drug resistance and virulence genes between different strains of *Staphylococcus* as much as possible.

In general, *S. sciuri* is a highly pathogenic bacterium for hybrid sturgeon. It spreads easily in the aquatic environment; therefore, attention should be paid to the cleanliness of breeding water and the disinfection of equipment during the aquaculture process. *S. sciuri* causes serious pathological damage to the liver, kidneys, and intestines, thus feeding management needs to be strengthened to improve the fish’s immunity. Targeted medication is recommended and the misuse of antibiotics is prohibited to avoid the development of drug-resistant bacteria or the transmission of drug-resistant genes to other species.

## 5 Conclusion

In this study, a strain of bacteria named D-59 was isolated from infected hybrid sturgeon and was confirmed as *Staphylococcus sciuri* using 16S rRNA gene, morphological observation, and bacterial biochemical identification. Analysis of changes in the gut microbiota of diseased sturgeon revealed that *S. sciuri* infection resulted in disruption of gut homeostasis, and a reduction in the abundance of gut commensal bacteria. By observing the histopathological changes following bacterial infection and the measurement of serological parameters, and comparing them with the results of previous studies, we showed that the D-59 strain caused severe damage to the liver tissue of animals, accompanied by persistent hemorrhage and a decrease in non-specific immune defenses. Drug sensitivity tests showed that D-59 is sensitive to florfenicol, enrofloxacin, neomycin sulfate, doxycycline, ampicillin, penicillin, gentamicin, minocycline, tetracyclines, and amikacin. Overall, the results of the present study provide a scientific basis for the etiology, diagnosis, and treatment of diseases caused by *S. sciuri* in cultured sturgeon.

## Data availability statement

The datasets presented in this study can be found in online repositories. The names of the repository/repositories and accession number(s) can be found below: https://www.ncbi.nlm.nih.gov/genbank/, SRR20887062

## Ethics statement

The animal study was reviewed and approved by the Animal Experimental Ethical Inspection of Laboratory Animal Centre, Yangtze River Fisheries Research Institute, Chinese Academy of Fishery Sciences.

## Author contributions

MZ and MX conceived and designed the study, performed the data collection, analysis, statistical analysis, and wrote the manuscript. ZX and WL conducted the software analysis and literature review. FX and NJ conducted the animal management and sample collections. YM and YF performed the microbial analysis, immunity analysis, and literature review. XL and YZ contributed to acquisition of funding, conceptualization, writing - review & editing, and supervision. All authors contributed to the article and approved the submitted version.

## Funding

This research was funded by the National Key Research Development Program of China, grant number 2019YFD0900105, the Central Public-interest Scientific Institution Basal Research Fund, grant number 2020TD44, and the National Freshwater Aquatic Germplasm Resource Center(FGRC18537).

## Conflict of interest

The authors declare that the research was conducted in the absence of any commercial or financial relationships that could be construed as a potential conflict of interest.

## Publisher’s note

All claims expressed in this article are solely those of the authors and do not necessarily represent those of their affiliated organizations, or those of the publisher, the editors and the reviewers. Any product that may be evaluated in this article, or claim that may be made by its manufacturer, is not guaranteed or endorsed by the publisher.
